# Functional imaging for assessing regional lung ventilation in preclinical and clinical research

**DOI:** 10.3389/fmed.2023.1160292

**Published:** 2023-05-16

**Authors:** Dipan Karmali, Mudiaga Sowho, Sonali Bose, Jackson Pearce, Vickram Tejwani, Zuzana Diamant, Keerthi Yarlagadda, Erick Ponce, Nina Eikelis, Tamas Otvos, Akram Khan, Michael Lester, Andreas Fouras, Jason Kirkness, Trishul Siddharthan

**Affiliations:** ^1^Division of Pulmonary and Critical Care, Leonard M. Miller School of Medicine, University of Miami, Coral Gables, FL, United States; ^2^Division of Pulmonary and Critical Care Medicine, Johns Hopkins School of Medicine, Baltimore, MD, United States; ^3^Division of Pulmonary and Critical Care, Icahn School of Medicine, Mount Sinai, NY, United States; ^4^School of Medicine, Medical University of South Carolina, Charleston, SC, United States; ^5^Respiratory Institute, Cleveland Clinic, Cleveland, OH, United States; ^6^Department of Microbiology Immunology and Transplantation, KU Leuven, Catholic University of Leuven, Leuven, Belgium; ^7^Department of Respiratory Medicine and Allergology, Institute for Clinical Science, Skane University Hospital, Lund University, Lund, Sweden; ^8^Department Clinical Pharmacy and Pharmacology, University of Groningen, University Medical Center Groningen, Groningen, Netherlands; ^9^4DMedical, Los Angeles, CA, United States; ^10^Division of Pulmonary and Critical Care, Oregon Health and Science University, Portland, OR, United States; ^11^Department of Pulmonary and Critical Care Medicine, Vanderbilt Medical Center, Nashville, CA, United States

**Keywords:** functional MRI, 4DCT, functional lung imaging, velocimetery, ventilation heterogeneity

## Abstract

Dynamic heterogeneity in lung ventilation is an important measure of pulmonary function and may be characteristic of early pulmonary disease. While standard indices like spirometry, body plethysmography, and blood gases have been utilized to assess lung function, they do not provide adequate information on regional ventilatory distribution nor function assessments of ventilation during the respiratory cycle. Emerging technologies such as xenon CT, volumetric CT, functional MRI and X-ray velocimetry can assess regional ventilation using non-invasive radiographic methods that may complement current methods of assessing lung function. As a supplement to current modalities of pulmonary function assessment, functional lung imaging has the potential to identify respiratory disease phenotypes with distinct natural histories. Moreover, these novel technologies may offer an optimal strategy to evaluate the effectiveness of novel therapies and therapies targeting localized small airways disease in preclinical and clinical research. In this review, we aim to discuss the features of functional lung imaging, as well as its potential application and limitations to adoption in research.

## Introduction

Several pulmonary diseases can affect the lungs non-uniformly, particularly in the early stages of disease (1–5). This may result in heterogenous ventilation and regional ventilation deficits, which has been associated with deleterious respiratory health outcomes (6). Initial functional testing modalities, like spirometry or static computed tomography (CT) scans, are unable to assess these regional differences in single-breath ventilation. Additionally, many existing modalities that assess ventilation heterogeneity require the administration of expensive radioisotopes and technical radiographic approaches precluding widespread use in clinical practice.

Spirometry, the most common test of lung function, often done with body plethysmography, quantifies global airflow and lung volumes, but is limited in detection of specific regional deficiencies (7–9). Alternatively, chest x-rays and CT scans are useful for identifying structural abnormalities of the lung, but only at a specific time point during the respiratory cycle (10–12). Over the past two decades, advances in CT image processing and magnetic resonance imaging (MRI), have produced the potential for extending the use of imaging beyond anatomic visualization to non-invasive evaluation of regional lung physiological function across the respiratory cycle (13–15). Additionally, X-ray velocimetry (XV) is a non-invasive fluoroscopy based image processing technique that uses lung tissue motion to determine spatial lung ventilation (16). This article aims to provide a brief overview and comparison of four dynamic imaging modalities, recognizing their potential applications in the field and identifying barriers to implementation in clinical practice.

### Xenon computed tomography

Xenon is an inert, noble gas that has been used as an inhalation contrast agent for functional lung imaging. Xenon-133 is advantageous because of its similar x-ray absorption characteristics as iodine, making it a useful inhalation contrast agent (17). Additionally, it has poor solubility in blood and tissue after inhalation, a longer half-life compared to oxygen-15 and nitrogen-13, and displays pulmonary function and disease better than krypton-81 (18, 19).

Xenon computed tomography was introduced in the 1980s as a functional imaging tool for measuring regional pulmonary ventilation. This imaging modality involves a wash-in phase, where the subject inhales a xenon and oxygen gas mixture over a short period of time. This is followed by a period of inhaling a high oxygen concentration while the radioisotope is exhaled or systemically absorbed, termed the washout phase. Xe CT scans can capture either at the beginning and end of the wash-in phase under a single-breath (static), or periodically over the course of multiple breaths throughout the wash-in and out phases (dynamic). Images are subsequently processed into a three-dimensional distribution map whereby areas of poor ventilation can be qualitatively and quantitatively assessed. Many variations of this procedure exist, and studies continue to optimize gas mixture concentrations and the timing of the wash-in and washout phases to better visualize regional ventilation, overall pulmonary function loss, and dynamic changes in the lung (19–21).

### 4D computed tomography

Diverse methods for volumetric are described in the literature; however, this has broadly been described using either paired (inhale/exhale) breath-hold CT (BHCT) or 4-Dimensional CT (4DCT) and comprising three computational steps: (1) lung volume delineation (segmentation), (2) measurement of lung motion through deformable image registration (DIR) or non-DIR methods, and (3) algorithmic calculation of surrogate measures for regional ventilation.

With respect to 4DCT algorithms, the most common algorithms for calculating ventilation metrics were evaluation of lung volume changes using CT intensity or Hounsfield Unit values (CTV-HU) and deformation vectors (Jacobian determinant; CTV-JAC), although variants and other methods were also reported in the literature. These images are then reconstructed, pairing images from the inhalation and exhalation phase thereby producing measurements for lung volume and ventilation (21). Through equations that take into account tidal volumes and respiratory variation, ventilation maps are produced, identifying regions of varying ventilation while reducing motion artifact (22, 23). Global and lobar ventilation 4D CT has been demonstrated to agree with other imaging modalities, like ventilation-perfusion images and ^129^Xe hyperpolarized MRI (24).

With respect to CTV algorithms, the most common algorithms for calculating ventilation metrics were evaluation of lung volume changes using CT intensity or Hounsfield Unit values (CTV-HU) and deformation vectors (Jacobian determinant; CTV-JAC), although variants and other methods were also reported in the literature. Overall, there was no clear evidence that one method was superior to another.

### Hyperpolarized gas magnetic resonance

MRI utilizes hydrogen atoms, abundant in soft tissue throughout the human body, and their associated magnetic environment to generate images that demonstrate detailed differences between various types of soft tissues (25, 26). Though useful in many clinical scenarios, MRI has traditionally offered limited tissue signaling of the lung parenchyma and airspace given low proton density in the lungs (27). However, the use of noble gas contrasts agents (similar to Xe CT), such as helium-3 (^3^He) or xenon-129 (^129^Xe) can help circumvent the relative lack of proton signal in the airspace, better capturing images of the lung tissue (28). Gaseous agents like ^3^He and ^129^Xe are hyperpolarized through spin exchange optical pumping (SEOP) to enhance their MR signaling properties (26, 29). When these gaseous agents are applied to MRI, the technique is known as hyperpolarized gas MR and offers a more robust evaluation of pulmonary anatomy and function, providing a dynamic look at ventilation of the airspace and perfusion of the parenchyma over the course of the respiratory cycle (27).

### X-ray velocimetry

X-ray velocimetry (XV) is a functional lung imaging technology that provides non-invasive quantification of regional ventilation during the respiratory cycle. XV technology is based on the principle of Particle Image Velocimetry (PIV), which allows to study the motion of particles within a medium. In practical terms, XV involves acquisition of fluoroscopic images over one complete and continuous tidal breath, which is captured at five angles, while the same center of rotation is maintained. The limited angles required for image acquisition allow for a significantly lower radiation dose to the patient compared to standard CT protocols (30).

**Figure 1:**
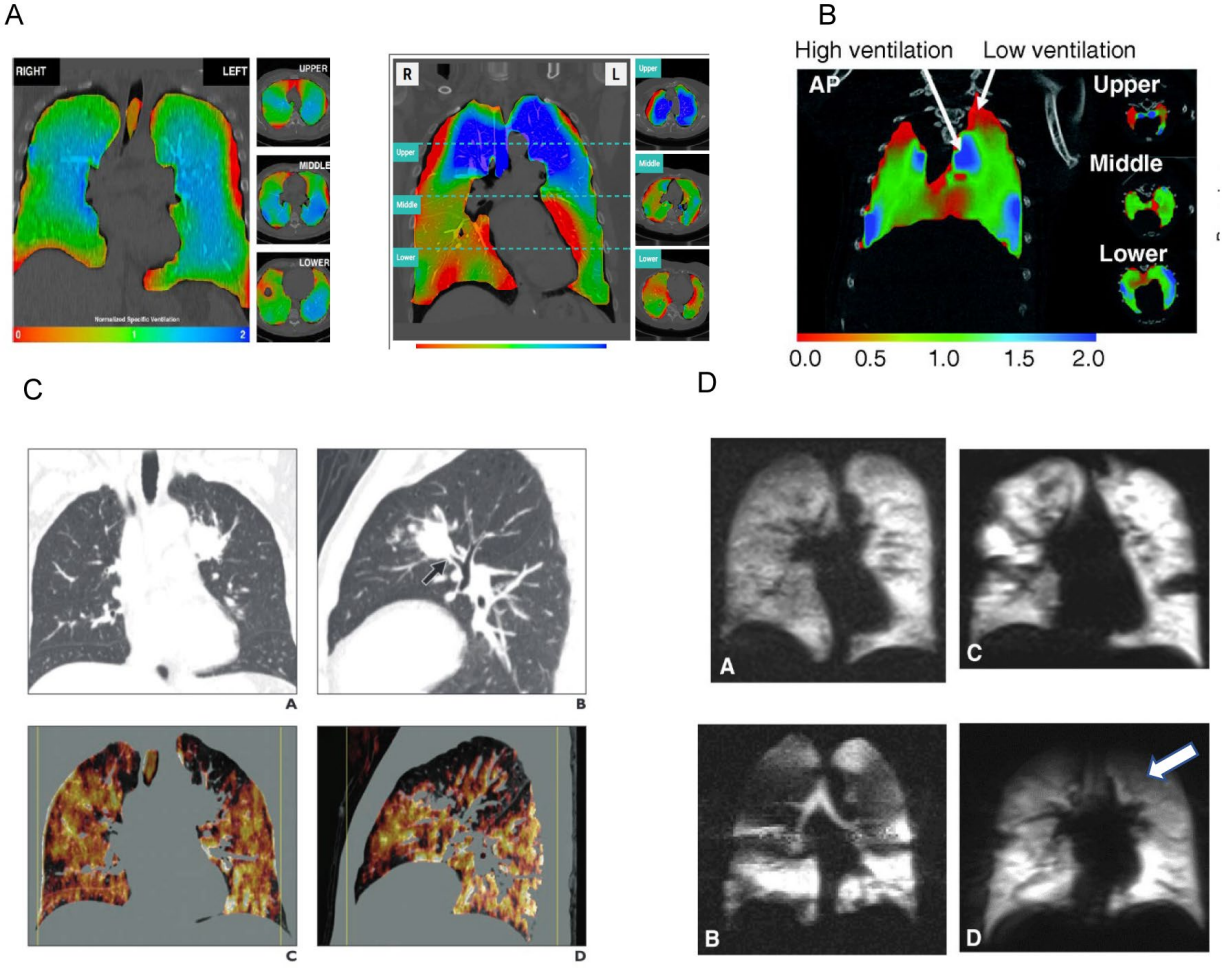
Examples of radiographically derived ventilation. Panel **(A)** shows XV analysis of regional lung ventilation using a color scale (red represents areas with decreased ventilation) (31). Panel **(B)** represents 4D CT imaging which shows regional areas of high ventilation and areas of low ventilation (red represents areas with decreased ventilation). Reprinted with permission of the American Thoracic Society. Copyright © 2023 American Thoracic Society. All rights reserved. The American Journal of Respiratory Cell and Molecular Biology is an official journal of the American Thoracic Society. (32). Panel **(C)** shows Xenon CT imaging with heterogenous distribution of Xenon due to this patient’s emphysematous disease. Ventilation defects are noted in the left upper lobe (white arrow). Adapted from the American Journal of Roentgenology, Copyright © 2014, copyright owner as specified in the American Journal of Roentgenology. (19). Panel **(D)** shows function MRI images with hyperpolarization of ^3^He/^129^Xe. Regional hypoventilation is noted by white arrows (33).

The analysis of the acquired images involves measuring tissue expansion over the duration of a tidal breath. Tissue expansion is then used to calculate regional ventilation at every location within the lung with several derived parameters. The information additionally enables spatial detection of regional ventilation defects and correlation to underlying lung structure (Figure 1).

## Applications of functional lung imaging

Early and accurate detection of respiratory disease remains an important goal in risk reduction and early treatment to prevent disease progression. Diagnosis and monitoring of lung disease relies primarily on functional measurements such as spirometry to assess the extent of functional impairment (8, 9), and imaging techniques such as X-rays or CT scans to identify underlying anatomic abnormalities (10, 11). Functional lung imaging has the potential to serve as a biomarker for respiratory disease in pre-clinical and clinical investigation over the past decade.

### Preclinical studies

#### Xenon CT

Few studies incorporating Xenon CT in animal models have been conducted. A murine study was able to successfully use continuous CT acquisition with Xe contrast to measure regional ventilation (34). A canine study demonstrated the decreased need to account for redistribution and recirculation of xenon as confounding variables in ventilation measurements (35). Finally, other studies have focused on integrating ventilation with perfusion in a single scan. For example, one research group studied pig models to measure ventilation with Xenon inhalation contrast and perfusion with gadolinium contrast, using a single CT image (36).

#### 4DCT

4DCT has been applied to evaluate ventilatory and perfusion defects in swine models with lung injury either from radiation for lung cancer or mechanical stress from mechanical ventilation. Wuschner et al. developed a specific swine model and correlated with human subjects to determine optimal biomarkers to monitor ventilatory changes after radiation therapy (37). Swine models have demonstrated dose dependent radiation correlating with decreased lung function (38). Separately, 4DCT was used to determine optimal lung-protective ventilation (39). Finally, Boehme et al. has used this mode in porcine models to quantify recruit and de-recruitment atelectasis, which contributes to ventilator-induced lung injury (40, 41).

#### Hyperpolarized gas MRI

Many pre-clinical studies have used hyperpolarized gas MRI to help elucidate the dynamic process by which gas diffusion and transport occurs and further understand the pathophysiology of pulmonary diseases (42). Mouse models studies have included hyperpolarized gas MRI to map the lungs anatomy and optimize quantitative measures of ventilation and diffusion (43). Similarly, mechanically ventilated, healthy rabbit models have utilized this technique with multi-breath wash-ins to describe ventilation heterogeneity (44). These techniques have proven useful in identifying ventilatory functional defects in diseased mice models, like emphysema (45). Additionally, hyperpolarized gas MRI has demonstrated longitudinal changes in the lung microenvironment in lung cancer models, creating a metric for treatment efficacy (46). Rodent models of pulmonary hypertension (PH) have been used to determine ventilation defects and diffusion impairments to monitor disease progression through non-invasive means (47). Hyperpolarized gas MRI has also been used in rat models for bronchopulmonary dysplasia to describe distal airway anatomy and in models for radiation lung injury to detect regional ventilation changes (48).

One limitation of prior models using hyperpolarized gas MR imaging is the necessity for well-controlled tidal volumes and timing of breaths. Although this is an approximate simulation for mechanically ventilated patients, these requirements limit the potential scope of use for hyperpolarized gas MR in free-breathing animal models. Recent studies have documented potential methods for obtaining functional lung imaging in free-breathing animal models. Loza et al. utilized a free-breathing murine model to assess imaging techniques that capture gas-phase images, measuring regional fractional ventilation, and dissolved-phase images, measuring gas uptake and distribution (26). These measurements provide a deeper evaluation of pulmonary function than traditional PFTs.

#### X ray velocimetry

As almost all lung pathologies are associated with regional changes in airflow throughout the lungs, it is essential to detect these functional changes in all locations during the entire respiratory cycle (32). The application of XV technology to quantify regional lung ventilation in animal models was first described in mice exposed to bleomycin, a well characterized experimental disease model that results in progressive lung injury (49). Regional maps of lung tissue motion revealed not only heterogeneity of normal lung ventilation but also aberrant airflow/motion as a result of bleomycin exposure (49). In another study, XV technology was applied to investigate lung dynamics in β-ENaC-overexpressing mice, a well-established model of lung disease (50). Overexpression of the epithelial Na + channel (ENaC) in the conducting airways causes airway surface liquid depletion and increased mucus concentration similar to that observed in human cystic fibrosis (*CF*) disease (51). Results of the study demonstrated marked heterogeneous lung function in β-ENaC transgenic mice compared to wild-type littermate controls (50).

### Clinical studies

#### Xenon CT

Most clinical studies have focused on applying Xe CT to lung disease populations and evaluating their degree of ventilation changes. For example, Xenon ventilation Dual Energy CT (DECT) has been used to visualize and quantify significant differences in ventilation between COPD and asthma-COPD overlap syndrome (52). Another study found regional ventilation changes and pulmonary function loss in chronic smokers, using Xe CT (18, 53). Investigating COPD patients, Xe CT has demonstrated qualitative ventilation changes similar to PFTs (54, 55).

Other studies have shown that Xe CT may be useful in diagnosing ventilation changes in other clinical contexts. One study identified additional ventilatory diagnostic information with Xe CT among mechanically ventilated patients, like discriminating bullae from pneumothorax, airway-to-pneumothorax fistula, and airway-to-mediastinum fistula (56). Another study was able to use Xe CT to identify ventilation and perfusion defects in patients suspected of pulmonary embolism (57). Additionally, using a Xe CT with methacholine and salbutamol inhalation challenge helped better visualize dynamic airflow changes in asthmatic patients. Finally, Xe CT has been used to effectively evaluate pulmonary function changes in patients surgically treated for NSCLC (58).

#### 4D CT and breath hold CT

Similarly to pre-clinical trials, there are limited, but ongoing, clinical trials that are focused on using 4D CT to preserve functional regions of lung ventilation for patients treated with radiation therapy for lung cancer. One ongoing phase 2 trial demonstrates 4D CT-guided ventilation as a useful clinical application in functional avoidance in patients with at least grade 2 radiation pneumonitis (59). Similar clinical trials are being conducted globally to optimize radiation therapy in lung cancer patients, like understanding lung ventilation physiology in chest radiation treatment, while still decreasing radiation dose administration, reducing tumor burden, and preserving ventilation (60, 61).

#### Hyperpolarized gas MRI

Hyperpolarized gas MRI is utilized in a variety of pulmonary pathologies, offers a more dynamic evaluation of pulmonary function, and lacks ionizing radiation (27). Roos et al. provides a review of the current and potential clinical applications of hyperpolarized gas MRI (62) Hyperpolarized gases through MR imaging have been used for the past 2 decades to detect and visualize abnormalities in the thoracic region. Presently, ventilation imaging provides helpful information about ventilation defects and diffusion-weighted imaging that can be utilized to calculate the apparent diffusion coefficient to differentiate between normal and enlarged airspaces. Future applications, as discussed above, include generating standardized, validated measurements in multi-centered trials, which would provide normal range values. Additionally, further information is needed to interpret these measurements and define its relationship with the pathophysiological process of lung diseases (33).

The ability to obtain functional information with a gaseous agent like ^129^Xe about diffusion and gas exchange would provide more useful information about pulmonary function than what is obtained with current pulmonary function testing, like spirometry. As an example of a possible clinical application, Lin et al. found that parameters measured with ^129^Xe hyperpolarized gas MR in pediatric asthmatics correlated with asthma severity and corticosteroid use (63). Similarly, hyperpolarized gas MR suggested a higher sensitivity than spirometry after bronchodilator inhalation in asthma patients (64). As hyperpolarized gas MR imaging gains attention for its potential clinical usefulness, there will be further understanding of the structure–function relationship in pulmonary diseases and their pathophysiological processes (65, 66). This could create a significant advantage over other imaging modalities in early disease detecting, monitoring, and phenotyping (33, 67).

#### XV technology

To date, no clinical studies related to XV have been published, though as the ventilation heterogeneity may occur over multiple breaths the application in clinical models could allow for breath-by-breath analysis.

## Promises and limitations of functional imaging

Although functional lung imaging has the potential to better characterize ventilatory disease and heterogeneity in ventilation within the lung, most of the imaging modalities have been applied within experimental settings and only recently have been included in clinical studies. Imaging modalities, such as Xe CT, 4DCT, and hyperpolarized gas MRI, are often cost-prohibitive for large clinical studies, requiring expensive isotopes and specialized equipment. Additionally, these studies represent different amounts of ionizing radiation exposure, limiting longitudinal assessments in some modalities (Table 1). More research needs to be done to optimize inhalation contrast delivery protocols and identify the utility of Xe CT, 4DCT, and hyperpolarized gas MRI in other acute and chronic disease processes that cause ventilation changes. Although XV offers a potentially lower cost assessment of ventilation compared to both Xe CT and hyperpolarized gas MRI, it is currently an experimental application with several limitations to adoption in clinical research. It is unclear whether displacement of lung tissue is truly a proxy of ventilation. Emphysematous areas of the lung will expand and contract with chest wall motion; however, these areas do not participate in gas exchange.

**Table 1:** Adult effective dose estimated range.

Adult effective dose estimate range	Example examinations
0 mSv	He MRI
<0.1 mSv	Chest radiograph; Hand radiographs
0.1–1 mSv	Pelvis radiographs, Mammography
0.2–2 mSv	Chest X-ray Velocimetry
1–10 mSv	Thoracic CT with IV contrast, Nuclear medicine bone scan
10–30 mSv	Thoracic CT without and with contrast; Whole body PET/CT
30–100 mSv	CTA chest abdomen and pelvis with contrast; Transjugular intrahepatic portosystemic shunt placement

Ongoing clinical trials, including multi-center studies, continue to evaluate healthy patients and patients with various lung diseases. This information will help generate reference ranges for regional ventilation that can be used to support clinical decision making. Future directions to assess the additive clinical benefit of functional lung imaging will require inclusion of cohort studies that further elucidate natural histories of disease and monitor therapeutic response to treatment.

## Conclusion

Innovations in imaging techniques, including functional lung imaging, show promise and the potential to improve our current assessment of lung function in preclinical and clinical models, as well as for clinical applications. To achieve robustness and precision, it is likely that future pulmonary function testing will incorporate these modalities to detect specific regional deficiencies in addition to characterizing global lung function. Such an approach will generate clinical information that will ultimately trigger a new era of pulmonary disease classification, patient monitoring and improved personalized care. Research is now ongoing to determine the utility of functional lung imaging in characterizing lung function in the clinical setting, as well as predicting morbidity in different kinds of pulmonary diseases.

## Author contributions

DK, MS, JP, JK, and TS performed initial writing of manuscript. SB, VT, KY, EP, NE, TO, AK, ML, and AF provided revisions to initial draft. All authors approved the final manuscript.

## Conflict of interest

NE, TO, AF, and JK, are employed by 4D Medical.

The remaining authors declare that the research was conducted in the absence of any commercial or financial relationships that could be construed as a potential conflict of interest.

## Publisher’s note

All claims expressed in this article are solely those of the authors and do not necessarily represent those of their affiliated organizations, or those of the publisher, the editors and the reviewers. Any product that may be evaluated in this article, or claim that may be made by its manufacturer, is not guaranteed or endorsed by the publisher.

## References

[1] Bates JHT. Lung mechanics. Cambridge: Cambridge University Press (2009).

[2] Suga K Tsukuda T Awaya H Takano K Koike S Matsunaga N. Impaired respiratory mechanics in pulmonary emphysema: Evaluation with dynamic breathing MRI. J Magn Reson Imaging. (1999) 10:510–07. doi: 10.1002/(SICI)1522-2586(199910)10:4<510::AID-JMRI3>3.0.CO;2-G, PMID: 10508317

[3] Suga K Nishigauchi K Kume N Koike S Takano K Matsunaga N. Regional ventilatory evaluation using dynamic SPET imaging of xenon-133 washout in obstructive lung disease: an initial study. Eur J Nucl Med. (1995) 22:220–6. doi: 10.1007/BF01081516, PMID: 7789394

[4] O’Riordan TG Iacono A Keenan RJ Duncan SR Burckart GJ Griffith BP. Delivery and distribution of aerosolized cyclosporine in lung allograft recipients. Am J Respir Crit Care Med. (1995) 151:516–1. doi: 10.1164/ajrccm.151.2.7842214, PMID: 7842214

[5] Brown JS Zeman KL Bennett WD. Regional deposition of coarse particles and ventilation distribution in healthy subjects and patients with cystic fibrosis. J Aerosol Med. (2001) 14:443–4. doi: 10.1089/08942680152744659, PMID: 11791685

[6] Barden RP. Radiological Diagnosis of Nonuniform Pulmonary Ventilation. Radiology. (1966) 86:417–07. doi: 10.1148/86.3.417, PMID: 5931762

[7] Liou TG Kanner RE. Spirometry. Clin Rev Allergy Immunol. (2009) 37:137–2. doi: 10.1007/s12016-009-8128-z, PMID: 19347610

[8] MacIntyre NR. The future of pulmonary function testing. Respir Care. (2012) 57:154–4. doi: 10.4187/respcare.01422, PMID: 22222134

[9] Ruppel GL Enright PL. Pulmonary Function Testing. Respir Care. (2012) 57:165–5. doi: 10.4187/respcare.01640 22222135

[10] Bankier AA Madani A Gevenois PA. CT quantification of pulmonary emphysema: assessment of lung structure and function. Crit Rev Comput Tomogr. (2002) 43:397–5. doi: 10.1080/10408370290807669, PMID: 12521149

[11] Raju S Ghosh S Mehta AC. Chest CT Signs in Pulmonary Disease. Chest. (2017) 151:1356–74. doi: 10.1016/j.chest.2016.12.033, PMID: 28212835

[12] Thakur SK Singh DP Choudhary J. Lung cancer identification: a review on detection and classification. Cancer Metastasis Rev. (2020) 39:989–8. doi: 10.1007/s10555-020-09901-x, PMID: 32519151

[13] Mirsadraee S van Beek EJR. Functional Imaging. Clin Chest Med. (2015) 36:349–3. doi: 10.1016/j.ccm.2015.02.014, PMID: 26024609

[14] Simon BA. Regional Ventilation and Lung Mechanics Using X-Ray CT1. Acad Radiol. (2005) 12:1414–22. doi: 10.1016/j.acra.2005.07.009, PMID: 16253853

[15] Simon BA Kaczka DW Bankier AA Parraga G. What can computed tomography and magnetic resonance imaging tell us about ventilation? J Appl Physiol. (2012) 113:647–7. doi: 10.1152/japplphysiol.00353.2012, PMID: 22653989 PMC3774257

[16] Dubsky S Jamison RA Irvine SC Siu KKW Hourigan K Fouras A. Computed tomographic x-ray velocimetry. Appl Phys Lett. (2010) 96:023702. doi: 10.1063/1.3285173, PMID: 34750782

[17] Meyer J Hayman L Yamamoto M Sakai F Nakajima S. Local cerebral blood flow measured by CT after stable xenon inhalation. Am J Roentgenol. (1980) 135:239–1. doi: 10.2214/ajr.135.2.239, PMID: 6773321

[18] Ohno Y Fujisawa Y Takenaka D Kaminaga S Seki S Sugihara N. Comparison of Xenon-enhanced area-detector CT and krypton ventilation SPECT/CT for assessment of pulmonary functional loss and disease severity in smokers. Am J Roentgenol. (2018) 210:W45–53. doi: 10.2214/AJR.17.18709, PMID: 29220212

[19] Kong X Sheng HX Lu GM Meinel FG Dyer KT Schoepf UJ. Xenon-enhanced dual-energy CT lung ventilation imaging: techniques and clinical applications. Am J Roentgenol. (2014) 202:309–7. doi: 10.2214/AJR.13.11191, PMID: 24450670

[20] Hoffman EA. Computed tomography studies of lung ventilation and perfusion. Proc Am Thorac Soc. (2005) 2:492–8. doi: 10.1513/pats.200509-099DS, PMID: 16352755 PMC2713338

[21] Jahani N Choi S Choi J Iyer K Hoffman EA Lin C-L. Assessment of regional ventilation and deformation using 4D-CT imaging for healthy human lungs during tidal breathing. J Appl Physiol. (2015) 119:1064–74. doi: 10.1152/japplphysiol.00339.2015, PMID: 26316512 PMC4816408

[22] Keall PJ Starkschall G Shukla H Forster KM Ortiz V Stevens CW. Acquiring 4D thoracic CT scans using a multislice helical method. Phys Med Biol. (2004) 49:2053–67. doi: 10.1088/0031-9155/49/10/015, PMID: 15214541

[23] Rietzel E Pan T Chen GTY. Four-dimensional computed tomography: Image formation and clinical protocol. Med Phys. (2005) 32:874–9. doi: 10.1118/1.1869852, PMID: 15895570

[24] Vinogradskiy Y. CT-based ventilation imaging in radiation oncology. BJR|Open. (2019) 1:20180035. doi: 10.1259/bjro.20180035, PMID: 33178925 PMC7592480

[25] Brian Dale PM Mark Brown P Richard SM. MRI: basic principles and Applications. 5th ed. New York: Wiley-Blackwell (2015).

[26] Loza LA Kadlecek SJ Pourfathi M Hamedani H Duncan IF Ruppert K. Quantification of Ventilation and Gas Uptake in Free-Breathing Mice With Hyperpolarized ^129^ Xe MRI. IEEE Trans Med Imaging. (2019) 38:2081–91. doi: 10.1109/TMI.2019.2911293, PMID: 30990426 PMC7268199

[27] Kern AL Vogel-Claussen J. Hyperpolarized gas MRI in pulmonology. Br J Radiol. (2018) 91:20170647. doi: 10.1259/bjr.20170647, PMID: 29271239 PMC5965996

[28] Mugler JP Altes TA. Hyperpolarized ^129^ Xe MRI of the human lung. J Magn Reson Imaging. (2013) 37:313–1. doi: 10.1002/jmri.23844, PMID: 23355432 PMC3558952

[29] Khan AS Harvey RL Birchall JR Irwin RK Nikolaou P Schrank G. Enabling Clinical Technologies for Hyperpolarized 129 Xenon Magnetic Resonance Imaging and Spectroscopy. Angew Chem Int Ed. (2021) 60:22126–47. doi: 10.1002/anie.202015200, PMID: 34018297 PMC8478785

[30] Goonan GW Fouras A Dubsky S. Array-source X-ray velocimetry. Opt Express. (2018) 26:935–07. doi: 10.1364/OE.26.000935, PMID: 29401982

[31] Vliegenthart R Fouras A Jacobs C Papanikolaou N. Innovations in thoracic imaging: CT, radiomics, <scp>AI</scp> and x-ray velocimetry. Respirology. (2022) 27:818–3. doi: 10.1111/resp.14344, PMID: 35965430 PMC9546393

[32] Asosingh K Frimel M Zlojutro V Grant D Stephens O Wenger D. Preclinical four-dimensional functional lung imaging and quantification of regional airflow: a new standard in lung function evaluation in murine models. Am J Respir Cell Mol Biol. (2022) 67:423–9. doi: 10.1165/rcmb.2022-0055MA, PMID: 35687482 PMC9564925

[33] Sharma M Wyszkiewicz PV Desaigoudar V Guo F Capaldi DP Parraga G. Quantification of pulmonary functional MRI: state-of-the-art and emerging image processing methods and measurements. Phys Med Biol. (2022) 67:22TR01. doi: 10.1088/1361-6560/ac9510, PMID: 36162409

[34] Lam WW Holdsworth DW Du LY Drangova M McCormack DG Santyr GE. Micro-CT imaging of rat lung ventilation using continuous image acquisition during xenon gas contrast enhancement. J Appl Physiol. (2007) 103:1848–56. doi: 10.1152/japplphysiol.00009.2007, PMID: 17690202

[35] Hoag JB Fuld M Brown RH Simon BA. Recirculation of Inhaled Xenon Does Not Alter Lung CT Density. Acad Radiol. (2007) 14:81–4. doi: 10.1016/j.acra.2006.10.012, PMID: 17178369 PMC1769338

[36] Sauter AP Hammel J Ehn S Achterhold K Kopp FK Kimm MA. Noël PB Perfusion-ventilation CT via three-material differentiation in dual-layer CT: a feasibility study. Sci Rep. (2019) 9:5837. doi: 10.1038/s41598-019-42330-7, PMID: 30967601 PMC6456734

[37] Wuschner AE Wallat EM Flakus MJ Shanmuganayagam D Meudt J Christensen GE. Radiation-induced Hounsfield unit change correlates with dynamic CT perfusion better than 4DCT-based ventilation measures in a novel-swine model. Sci Rep. (2021) 11:13156. doi: 10.1038/s41598-021-92609-x, PMID: 34162987 PMC8222280

[38] Wallat EM Wuschner AE Flakus MJ Christensen GE Reinhardt JM Shanmuganayagam D. Radiation-induced airway changes and downstream ventilation decline in a swine model. Biomed Phys Eng Express. (2021) 7:065039. doi: 10.1088/2057-1976/ac3197, PMID: 34670195 PMC8785227

[39] Herrmann J Gerard SE Shao W Hawley ML Reinhardt JM Christensen GE. Quantifying regional lung deformation using four-dimensional computed tomography: a comparison of conventional and oscillatory ventilation. Front Physiol. (2020) 11:e00014. doi: 10.3389/fphys.2020.00014, PMID: 32153417 PMC7044245

[40] Boehme S Toemboel FPR Hartmann EK Bentley AH Weinheimer O Yang Y. Markstaller K Detection of inspiratory recruitment of atelectasis by automated lung sound analysis as compared to four-dimensional computed tomography in a porcine lung injury model. Crit Care. (2018) 22:50. doi: 10.1186/s13054-018-1964-6, PMID: 29475456 PMC6389194

[41] Preissner M Murrie RP Pinar I Werdiger F Carnibella RP Zosky GR. High resolution propagation-based imaging system for in vivo dynamic computed tomography of lungs in small animals. Phys Med Biol. (2018) 63:08NT03. doi: 10.1088/1361-6560/aab8d2, PMID: 29565260

[42] Kadlecek S Friedlander Y Virgincar RS. Preclinical MRI Using Hyperpolarized 129Xe. Molecules. (2022) 27:8338. doi: 10.3390/molecules27238338, PMID: 36500430 PMC9738892

[43] Niedbalski PJ Cochran AS Akinyi TG Thomen RP Fugate EM Lindquist DM. Preclinical hyperpolarized ^129^ Xe MRI: ventilation and T _2_ * mapping in mouse lungs at 7 T using multi-echo flyback UTE. NMR Biomed. (2020) 33:e4302. doi: 10.1002/nbm.4302, PMID: 32285574 PMC7702724

[44] Hamedani H Kadlecek S Ruppert K Xin Y Duncan I Rizi RR. Ventilation heterogeneity imaged by multibreath wash-ins of hyperpolarized 3 He and 129 Xe in healthy rabbits. J Physiol. (2021) 599:4197–23. doi: 10.1113/JP281584, PMID: 34256417 PMC9186264

[45] Wakayama T Ueyama T Imai F Kimura A Fujiwara H. Quantitative assessment of regional lung ventilation in emphysematous mice using hyperpolarized 129Xe MRI with a continuous flow hyperpolarizing system. Magn Reson Imaging. (2022) 92:88–95. doi: 10.1016/j.mri.2022.05.017, PMID: 35654279

[46] Kimura A Utsumi S Shimokawa A Nishimori R Stewart NJ Kamada Y. Fujiwara H Inflammation during Lung Cancer Progression and Ethyl Pyruvate Treatment Observed by Pulmonary Functional Hyperpolarized 129Xe MRI in Mice. Contrast Media Mol Imaging. (2021) 2021:1–10. doi: 10.1155/2021/9918702, PMID: 34257627 PMC8261185

[47] Virgincar RS Nouls JC Wang Z Degan S Qi Y Xiong X. Quantitative 129Xe MRI detects early impairment of gas-exchange in a rat model of pulmonary hypertension. Sci Rep. (2020) 10:7385. doi: 10.1038/s41598-020-64361-1, PMID: 32355256 PMC7193602

[48] Fliss JD Zanette B Friedlander Y Sadanand S Lindenmaier AA Stirrat E. Hyperpolarized 129 Xe magnetic resonance spectroscopy in a rat model of bronchopulmonary dysplasia. Am J Phys Lung Cell Mol Phys. (2021) 321:L507–17. doi: 10.1152/ajplung.00612.2020, PMID: 34189953

[49] Fouras A Allison BJ Kitchen MJ Dubsky S Nguyen J Hourigan K. Altered lung motion is a sensitive indicator of regional lung disease. Ann Biomed Eng. (2012) 40:1160–9. doi: 10.1007/s10439-011-0493-0, PMID: 22189492

[50] Stahr CS Samarage CR Donnelley M Farrow N Morgan KS Zosky G. Quantification of heterogeneity in lung disease with image-based pulmonary function testing. Sci Rep. (2016) 6:29438. doi: 10.1038/srep29438, PMID: 27461961 PMC4962033

[51] Mall MA. ENaC inhibition in cystic fibrosis: potential role in the new era of CFTR modulator therapies. Eur Respir J. (2020) 56:2000946. doi: 10.1183/13993003.00946-2020, PMID: 32732328 PMC7758539

[52] Hwang HJ Lee SM Seo JB Lee JS Kim N Lee SW. Visual and Quantitative Assessments of Regional Xenon-Ventilation Using Dual-Energy CT in Asthma-Chronic Obstructive Pulmonary Disease Overlap Syndrome: A Comparison with Chronic Obstructive Pulmonary Disease. Korean J Radiol. (2020) 21:1104–13. doi: 10.3348/kjr.2019.0936, PMID: 32691546 PMC7371623

[53] Ohno Y Fujisawa Y Sugihara N Kishida Y Koyama H Seki S. Wash-in/wash-out phase xenon-enhanced area-detector CT (ADCT): utility for regional ventilation, pulmonary functional loss and clinical stage evaluations of smokers. Acta Radiol. (2019) 60:1619–28. doi: 10.1177/0284185119840647, PMID: 30997827

[54] Kyoyama H Hirata Y Kikuchi S Sakai K Saito Y Mikami S. Evaluation of pulmonary function using single-breath-hold dual-energy computed tomography with xenon. Medicine. (2017) 96:e5937. doi: 10.1097/MD.0000000000005937, PMID: 28099359 PMC5279104

[55] Kim M Doganay O Hwang HJ Seo JB Gleeson FV. Lobar Ventilation in Patients with COPD Assessed with the Full-Scale Airway Network Flow Model and Xenon-enhanced Dual-Energy CT. Radiology. (2021) 298:201–9. doi: 10.1148/radiol.2020202485, PMID: 33231530

[56] Hoegl S Meinel FG Thieme SF Johnson TRC Eickelberg O Zwissler B. Worsening respiratory function in mechanically ventilated intensive care patients: Feasibility and value of xenon-enhanced dual energy CT. Eur J Radiol. (2013) 82:557–2. doi: 10.1016/j.ejrad.2012.10.029, PMID: 23238360

[57] Zhang LJ Zhou CS Schoepf UJ Sheng HX Wu SY Krazinski AW. Dual-energy CT lung ventilation/perfusion imaging for diagnosing pulmonary embolism. Eur Radiol. (2013) 23:2666–75. doi: 10.1007/s00330-013-2907-x, PMID: 23760304

[58] Ohno Y Fujisawa Y Yoshikawa T Takenaka D Koyama H Hattori H. Inspiratory/expiratory xenon-enhanced area-detector CT: Capability for quantitative assessment of lung ventilation changes in surgically treated non-small cell lung cancer patients. Eur J Radiol. (2021) 136:109574. doi: 10.1016/j.ejrad.2021.109574, PMID: 33548852

[59] Vinogradskiy Y Castillo R Castillo E Schubert L Jones BL Faught A. Results of a Multi-Institutional Phase 2 Clinical Trial for 4DCT-Ventilation Functional Avoidance Thoracic Radiation Therapy. Int J Radiat Oncol Biol Phys. (2022) 112:986–5. doi: 10.1016/j.ijrobp.2021.10.147, PMID: 34767934 PMC8863640

[60] Li S Liu J Gao S Yin Y Zhang L Han Y. CT ventilation image-guided helical Tomotherapy at sparing functional lungs for locally advanced lung cancer: analysis of dose-function metrics and the impact on pulmonary toxicity. Radiat Oncol. (2023) 18:6. doi: 10.1186/s13014-022-02189-x, PMID: 36624537 PMC9830733

[61] Huang Y Ren G Xiao H Yang D Kong F Ho WY. Volumetric multiphase ventilation imaging based on four-dimensional computed tomography for functional lung avoidance radiotherapy. Med Phys. (2022) 49:7237–46. doi: 10.1002/mp.15847, PMID: 35841346

[62] Roos JE McAdams HP Kaushik SS Driehuys B. Hyperpolarized Gas MR Imaging. Magn Reson Imaging Clin N Am. (2015) 23:217–9. doi: 10.1016/j.mric.2015.01.003, PMID: 25952516 PMC4428591

[63] Lin NY Roach DJ Willmering MM Walkup LL Hossain MM Desirazu P. 129Xe MRI as a measure of clinical disease severity for pediatric asthma. J Allergy Clin Immunol. (2021) 147:2146–2153.e1. doi: 10.1016/j.jaci.2020.11.010, PMID: 33227317

[64] Marshall H Kenworthy JC Horn FC Thomas S Swift AJ Siddiqui S. Peripheral and proximal lung ventilation in asthma: Short-term variation and response to bronchodilator inhalation. J Allergy Clin Immunol. (2021) 147:2154–2161.e6. doi: 10.1016/j.jaci.2020.11.035, PMID: 33309743

[65] Gefter WB Lee KS Schiebler ML Parraga G Seo JB Ohno Y. Pulmonary functional imaging: Part 2—state-of-the-art clinical applications and opportunities for improved patient care. Radiology. (2021) 299:524–8. doi: 10.1148/radiol.2021204033, PMID: 33847518 PMC8165948

[66] Svenningsen S McIntosh M Ouriadov A Matheson AM Konyer NB Eddy RL. Reproducibility of Hyperpolarized 129Xe MRI Ventilation Defect Percent in Severe Asthma to Evaluate Clinical Trial Feasibility. Acad Radiol. (2021) 28:817–6. doi: 10.1016/j.acra.2020.04.025, PMID: 32417033

[67] Guiot J Vaidyanathan A Deprez L Zerka F Danthine D Frix A-N. A review in radiomics: making personalized medicine a reality via routine imaging. Med Res Rev. (2022) 42:426–07. doi: 10.1002/med.21846, PMID: 34309893

